# The Synergetic Effect of Cash Transfers for Families, Child Sensitive Social Protection Programs, and Capacity Building for Effective Social Protection on Children’s Nutritional Status in Nepal

**DOI:** 10.3390/ijerph14121502

**Published:** 2017-12-04

**Authors:** Andre M. N. Renzaho, Stanley Chitekwe, Wen Chen, Sanjay Rijal, Thakur Dhakal, Pradiumna Dahal

**Affiliations:** 1 Humanitarian and Development Studies, School of Social Sciences and Psychology, Western Sydney University, Locked Bag 1797, Penrith, NSW 2751, Australia; Wen.Chen@westernsydney.edu.au; 2 School of Public Health and Preventive Medicine, Monash University, The Alfred Centre, 99 Commercial Road, Melbourne, VIC 3004, Australia; 3 UNICEF (United Nations Children’s Fund) Nepal, UN House Pulchowk, P.O. Box 1187, Kathmandu, Nepal; schitekwe@unicef.org (S.C.); sarijal@unicef.org (S.R.); tdhakal@unicef.org (T.D.); pdahal@unicef.org (P.D.); 4 Faculty of Medical Statistics and Epidemiology, School of Public Health, Sun Yat-sen University, Guangzhou 510080, China

**Keywords:** child cash grant, social protection, Nepal, wasting, stunting, and underweight

## Abstract

Objective: The aim of this study was to evaluate the effectiveness of the synergetic effect of child sensitive social protection programs, augmented by a capacity building for social protection and embedded within existing government’s targeted resource transfers for families on child nutritional status. Design: A repeat cross-sectional quasi-experimental design with measures taken pre- (October–December 2009) and post- (December 2014–February 2015) intervention in the intervention and comparison district. The comparison district received standard social welfare services in the form of targeted resource transfers (TRTs) for eligible families. The intervention district received the TRTs plus a child cash payment, augmented by a capacity building for effective social protection outcomes. Propensity scores were used in difference-in-differences models to compare the changes over time between the intervention and control groups. **Results:** Propensity score matched/weighted models produced better results than the unmatched analyses, and hence we report findings from the radius matching. The intervention resulted in a 5.16 (95% CI: 9.55, 0.77), 7.35 (95% CI: 11.62, 3.08) and 2.84 (95% CI: 5.58, 0.10) percentage point reduction in the prevalence of stunting, underweight, and wasting among children under the age, respectively. The intervention impact was greater in boys than girls for stunting and wasting; and greater in girls than boys for underweight. The intervention also resulted in a 6.66 (95% CI: 2.13, 3.18), 11.40 (95% CI: 16.66, 6.13), and 4.0 (95% CI: 6.43, 1.78) percentage point reduction in the prevalence of stunting, underweight, and wasting among older children (≥24 months). No impact was observed among younger children (<24 months). Conclusions: Targeted resource transfers for families, augmented with a child sensitive social protection program and capacity building for social protection can address effectively child malnutrition. To increase the intervention effectiveness on younger children, the child cash payment amount needs to be revisited and closely embedded into infant and young child feeding initiatives, but also adjusted to equate to 20% of household expenditure or more to maximize the diversity of food available to young children.

## 1. Background

According to the World Health Organisation [1], children under the age of five years are among the most vulnerable to an impaired health environment, with approximately 5.9 million dying in 2015. Most of these deaths were preventable, with more than half of the child deaths closely linked to conditions that can be prevented and/or treated with access to simple, affordable interventions. However, the 2013 Lancet series on maternal and child malnutrition reported that child malnutrition (foetal growth restriction, suboptimum breastfeeding, stunting, wasting, and deficiencies of vitamin A and zinc) remains the leading cause of child death, accounting for 45% of all deaths among children under the age of five years [2]. In most low-income countries, underweight, stunting and wasting represent a serious problem with short and long term debilitating effects. For this reason, addressing child undernutrition has always been a priority in many international agendas, and lately political stimulation and commitment have been achieved through advocacy work by the Scaling Up Nutrition (SUN) Movement and the Zero Hunger Initiative.

For example, reducing the prevalence of child malnutrition, especially underweight among children under the age of five, was included as an indicator for Goal 1 (Eradicate extreme poverty and hunger) of the 2000 Millennium Development Goals (MDGs) [3]. This indicator was expanded in the 2015 Sustainable Development Goals (SDGs), with a stated target of ending all forms of malnutrition among children under five years of age by 2030, including reducing stunting, wasting and overweight as indicators for Goal 2 (End hunger, achieve food security and improved nutrition and promote sustainable agriculture, Target 3.4) of the SDG [4]. The 2012 World Health Assembly Resolution 65.6 that endorsed a comprehensive implementation plan on maternal, infant and young child nutrition; and accompanied with specified six global nutrition targets for 2025. These are 40% reduction in the number of children under-five who are stunted; 50% reduction of anaemia in women of reproductive age; 30% reduction in low birth weight; no increase in childhood overweight; increase the rate of exclusive breastfeeding in the first six months up to at least 50%; and reduce and maintain childhood wasting to less than 5%) [5,6].

Although the proportion of children under the age of five years who are malnourished has almost been halved between 1990 and 2015, the results were mixed. Regions that met the MDG target related to child underweight were Eastern Asia, Latin America, and the Caribbean, the Caucasus and Central Asia, Northern Africa, South-Eastern Asia and Western Asia, with the prevalence of underweight falling by only one-third in sub-Saharan Africa since 1990 [3]. However, the Southern Asia region recorded the highest underweight prevalence but the largest absolute decrease of a 22 percentage-point drop since 1990 [3].

Nepal is one of the Southern Asian countries to achieve the majority of the MDG targets on children and pregnant mothers [7]. Data from the Demographic Health Surveys suggest that the prevalence of stunting, underweight, and wasting have been decreasing, with the percentage of stunted children declining from 57% in 2001 to 41% in 2011, and to 36% in 2016 [8]. A similar downward trend was recorded for underweight, declining from 43% in 2001 to 29% in 2011, and to 27% in 2016. The observed downward trend during this period was in line with the MDG targets but still far off meeting the SDG targets of reducing child stunting and underweight. The trend in the prevalence of wasting has remained static over the last 16 years, estimated at 11% in 2001, 13% in 2006, 11% in 2011, and 10% in 2016 [8]. Despite the downtrend in the prevalence of child malnutrition observed in Nepal, the prevalence of wasting, underweight, and stunting is still high and can be classified as “serious” based on the World Health Organisation’s classification of the severity of child malnutrition [9] and still significantly higher than the world average [3]. Additionally, the downtrend achievement is unequally distributed and acute food shortages and high rates of child undernutrition continue to affect vulnerable women and children in the hills and mountains of the mid- and far-western regions [8]. For example, the prevalence of stunting is significantly higher in the rural areas than urban areas, in the mountain zones than non-mountain zones, among children of mothers with no education than those whose mothers have been to school [8]. The prevalence of wasting and underweight followed a similar pattern. In addition, the prevalence of child stunting and underweight increased with age, peaking at 44.5% and 30.7%, respectively, between 24 and 35 months. In contrast, the prevalence of wasting decreased with age, with high rates reported among infants (≥15% among children aged less than two years, peaking at 21.3% between six and eight months) [8].

The above data suggest that there is an urgent need to scale up intervention to improve infant and young child nutrition in Nepal. It is well documented that the period between conception and two years (“the first 1000 days”) represents a window of opportunity to address child malnutrition. For chronic states such as stunting and all associated pathology, the period represents the most responsive to interventions with a high likelihood of reversing it but little apparent recovery beyond 24 months of age [10]. However, there is a global consensus on the multi-sectoral to approaches to nutrition that integrates nutrition-sensitive and nutrition-specific intervention to address all form of child malnutrition and to accelerate progress. Nutrition-specific interventions address the immediate determinants of foetal and child nutrition: (i) adequate food and nutrient intake; and (ii) control of infectious diseases. They encompass exclusive breastfeeding promotion, complementary feeding, micronutrient supplementation and food fortification, maternal health and nutrition, dietary diversification, deworming, treatment of severe wasting, and the prevention and management of infectious diseases [11]. In contrast, nutrition-sensitive interventions address the underlying determinants: (i) food security; (ii) social and care behaviours at maternal, household, and community levels; and (iii) health environment and health services. The 2013 Lancet series on maternal and child malnutrition highlighted the impact of various individual nutrition-specific interventions on child and maternal nutrition status [12]. The series concluded that the synergetic effect between nutrition-specific and nutrition-sensitive interventions, and indeed between different types of nutrition-specific interventions, must be evaluated. Such programs need to be embedded into existing community structures and incorporate capacity-building of beneficiary communities to maximise sustainability [13].

Social protection is one form of nutrition-sensitive interventions addressing malnutrition indirectly through sectoral policies linked to underlying (household-level) and basic (community and state-level) causes of child malnutrition. Conceptually, defining social protection has taken various forms depending on the objectives of funding agencies, which vary widely, and type of the program beneficiaries. Therefore, there remains a lack of consensus on what constitutes “social protection”, but broadly it encompasses a variety of public and private social programs and initiatives that “provide income or consumption transfers to the poor, protect the vulnerable against livelihood risks and enhance the social status and rights of the marginalised; with the overall objective of reducing the economic and social vulnerability of poor, vulnerable and marginalised groups” [14].

According to Devereux and Sabates-Wheeler [14], four categories of social protection programs can be identified based on their functions. The first category is the protective function which guarantees relief from deprivation and recovery from shocks. Programs include social assistance to individual or families who are poor and unable to work and earn their livelihood; social welfare that include targeted resource transfers such as disability benefit, single-parent allowances, old age pension; and social services to vulnerable populations such as orphans and abandoned children or displaced people. The second category is the preventative function which focuses on averting deprivation through social insurance or social safety nets for economically vulnerable people including pensions, health insurance, maternity benefit and unemployment benefits, as well as informal mechanisms such as savings clubs and funeral societies. The third category is the promotive function which enhances income, income stabilisation, and consumption smoothing through microfinance; and capabilities through a range of livelihood-enhancing programmes such as school feeding programs. The last category is the transformative function which upholds social equity and inclusion of socially marginalised groups such as people with disabilities, victims of domestic violence or discrimination and abuses; and promotes empowerment and human rights through collective actions and legal or regulatory frameworks.

Social protection programs and child nutrition have rapidly moved up the policy agenda internationally. Social protection programs have been implemented in different forms with great impact on child nutrition outcomes, and they can be classified into two major rubrics [15]. The first rubric is cash-based social transfers in the form of regular, non-contributory payments of money to eligible individuals or households by the government or nongovernment organisations. They encompass cash transfers including unconditional and conditional (i.e., social contract with beneficiary households or individuals to fulfil responsibilities related to education, health, nutrition, or the provision of labour in compliance with a work requirement), cash-for-work/asset, labour-intensive public works; near-cash transfers such as value-based vouchers; and grants such as lump sum grant. The second rubric is in-kind social transfers including food transfers such as school feeding, take-home rations, targeted food distributions, food-for-work/asset, food-for-training, and preventive supplementary feeding; commodity vouchers such as food vouchers and other commodity vouchers; and asset and input transfers such as livestock transfer, agricultural input transfer, and asset transfer. Cash-based and in-kind social transfers can be universal or explicitly targeted to those identified as poor or vulnerable. Available evidence suggests a positive impact of cash-based and in-kind social transfers, making a significant contribution to addressing poverty and vulnerability amongst the poorest households in developing countries including improvements in nutrition, access to health care, and health status among beneficiaries, improvements in secondary school enrolment and attendance, reduction in child labour in rural areas, making important contributions to employment generation, and improvement in productive and financial asset accumulation [16,17]. Cash-based and in-kind social transfers have improved children’s nutritional status and anthropometric outcomes in Sub-Saharan Africa [17,18], Latin American countries [17,19,20,21,22,23,24,25], and Asia [17,26,27,28,29,30]. Overall, the findings suggest that pensions received by women have a large impact on wasting and stunting among girls, but have little effect on boys [18]. In contrast, no similar effect has been found for pensions received by men, suggesting that the effectiveness of government-sponsored social protection programs may depend on the gender and who receives the social transfer within the household [18].

Despite the above findings, the working mechanism of cash-based and in-kind social transfers on children’s nutritional status is not unequivocal. Firstly, the impact of cash-based transfer interventions has varied across settings, and it is not clear as what are the best ways to implement such programs [30]. Secondly, the effectiveness of different modalities of cash-based transfer interventions has varied according to the types of malnutrition, with little evidence of its long-term impact and its impact on wasting in young children in humanitarian aid settings [30]. Some studies have found that the duration of the program and the amount of cash given have the greatest effect [30]. However, other studies have shown no association between exposure to cash-based and in-kind social transfers and stunting in children [31]. Finally, studies that included children younger than 12 months of age at baseline reported the greatest benefit on height-based measures among children younger than two years when compared to older children exposed to the programme for one year [24,32].

All the above evidence suggests there is an urgent need to test the synergic effects of various social transfer programs on child malnutrition status in various settings. The effect of cash transfer programs is maximised if the eligibility for the targeted cash transfer programs is limited to the “window of opportunity” for nutrition impact while prioritising nutrition-related conditionalities and coordination with other key agencies and stakeholders [33]. Even doing so has still produced conflicting results, with some studies showing no overall association between program participation and growth in children 6 to 24 month of age [34], and no impact on stunting [33]. Where an impact was observed, it was found only among children younger than two years, compared with children aged 2 years or older. The aim of this study was to evaluate the effectiveness of the synergetic effect of child sensitive social protection programs, augmented by a capacity building for social protection and embedded within existing government’s targeted resource transfers for families on child nutritional status.

## 2. Methods

### 2.1. Evaluation Design and the Intervention

The evaluation was used a repeat cross-sectional quasi-experimental design with measures taken pre- (October–December 2009) and post- (December 2014–February 2015) intervention in the intervention community (Kalikot district) and comparison communities (Bajhang district). The quasi-experimental design was the most appropriate design because randomisation was impractical and the study aimed at minimising threats to ecological validity, hence providing adequate avenues for testing the effectiveness of community-based interventions in real-world settings [35].

The comparison district received government’s funded targeted resource transfers (TRTs) for families. The TRTs included senior citizens allowance for all persons aged 70+ (500 Nepalese rupees (NRs)/month), single women’s and widow allowance (NRs 500/month), disability allowance for all people with disability aged 16 years or older (NRs 1000/month for total disability and NRs 300/month for partial disability), endangered ethnicities allowance (all household members receive NRs 500/month), and maternity incentive scheme for pregnant women (NRs 500 in Tarai, NRs 1000 in Hills and NRs 1500 in mountains as transportation costs plus NRs 300 provided to health professionals and NRs 1000 reimbursement to facilities plus free delivery care).

**Figure 1:**
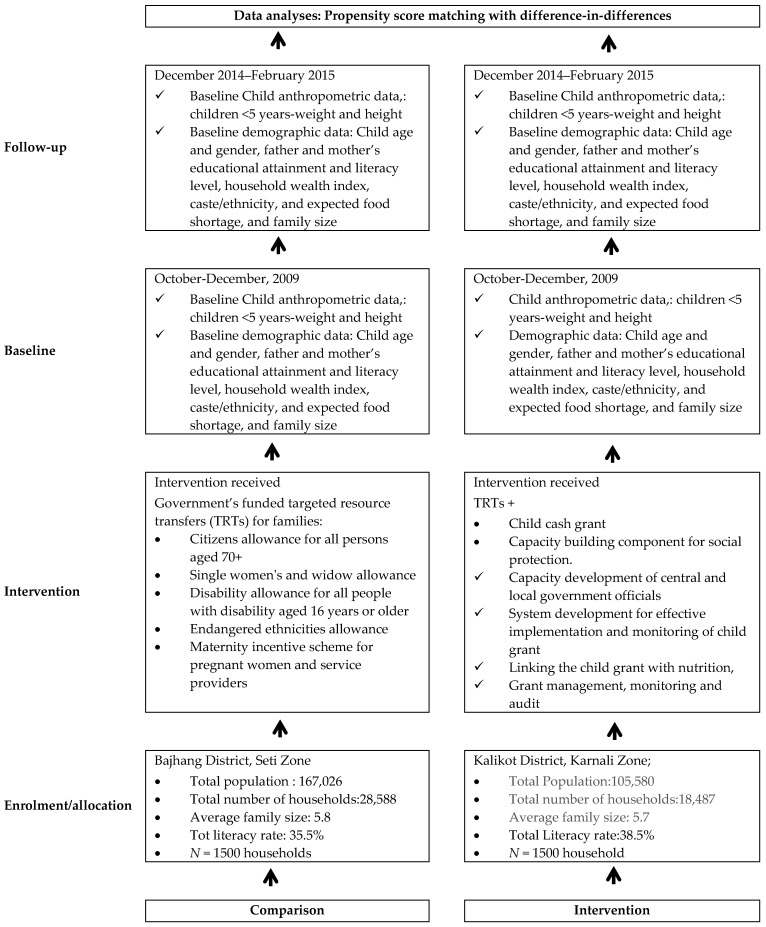
Flow diagram detailing the intervention implementation plan and data collection phases.

The intervention district received the TRTs, augmented with a child cash grant (CCG) program introduced in the Government of Nepal’s (GoN’s) 2009/2010 budget and a capacity building component for social protection (Figure 1). The CCG provides NRs 200 per month for up to two children for poor families with children under five in Karnali Zone (Kalikot, Jumla, Mugu, Humla and Dolpa) to complement existing social protection schemes for senior citizens, single women, endangered communities and people with disabilities. The GoN’s CCG is an unconditional cash transfer scheme in which allowances are provided to all eligible households. The CCG program has been supported and enhanced by the capacity building for social protection implemented by a UNICEF (United Nations Children’s Fund)/Nepal partnership program, whose aim has been to design and implement complementary interventions, partly funded by the Asian Development Bank through Japan Fund for Poverty Reduction (Table 1). The capacity building for social protection had four major components: (1) capacity development of central and local government officials; (2) system development for effective implementation and monitoring of child grant; (3) linking the child grant with nutrition; and (4) grant management, monitoring and audit. The Ministry of Federal Affairs and Local Development (the main executing agency) was responsible for the system development component and the Asia Development Bank together with the Ministry of Federal Affairs and Local Development and the Ministry of Health and Population were responsible for grant management component. The United Nations Children’s Fund (UNICEF) was responsible for implementation of the capacity development and linking CCG with nutrition and supported the GoN (Ministry of Federal Affairs and Local Development and Ministry of Health and Population) in implementing key strategies underpinning the intervention. These were:

(1) capacity building to enhance the capacity of local bodies in the project districts to deliver the child grant, through orientations for Village Development Committee (VDC) leaders, Traditional Healers and mothers/caretakers, and capacity-building for health workers and Female Community Health Volunteers (FCHVs) and VDC secretaries;
(2) enhancing networking between local bodies, health facilities and communities in the project districts to improve child nutrition;
(3) social behaviour change communication on child nutrition including the provision of nutrition-related counselling services;
(4) awareness raising for timely birth registration to identify all eligible households and about the availability of the CCG;
(5) assisting mothers and others caring for children to identify the best possible locally available food and encouraging them to use the CCG for nutritious foods and the improvement of the nutritional status of children; and
(6) improving the knowledge and skills of CCG beneficiaries in the areas of infant and young child feeding (IYCF) practices, hygiene, sanitation, and other key behaviours linked to child nutrition.

### 2.2. Sampling Strategy

The surveys were conducted using a two-stage cluster sampling method. The first stage involved identifying clusters (wards) within each district to be included in the study. All wards in each district were listed separately in alphabetical order by VDC. Using the 2011 population census data for each ward (cluster), a cumulative population for all wards was computed. From this cumulative list, the required number of clusters in each district was determined using the probability proportional to size sampling method.

In the second stage, households within the selected clusters were identified for inclusion in the study. A list of households in each selected ward was constructed with the help of the local leaders and UNICEF staff. From the list, a household was selected using a systematic sampling approach. Only households with at least one child under 60 months of age were eligible for the study. The sampling interval (X) was determined by dividing the total number of households in each ward with the expected sample size, and the first household to be surveyed was randomly selected by choosing a number between 1 and X. For each selected household, mothers/caretakers of children under five years of age volunteered to take part in the surveys, and the interview occurred outside the home, away from other household members. If the selected household was not inhabited, or there was no one at home, the closest neighbouring household was used for the survey. We sampled approximately 30 households per cluster in each selected district at baseline, midline and endline surveys. For clusters where the number of households was less than 25, the selected ward and its adjoining neighbour were merged and treated as a single cluster. In households with more than one child, only one child was randomly selected for enumeration. The study was approved by the Nepal Health Research Council Ethical Review Board (Approval No. 2071-12-18; Reg No. 29/2015).

### 2.3. Sample Size

The sample size calculation was primarily to detect meaningful levels of change in the study outcomes compared to the comparison group. We planned to sample only one child per household, hence an equal sample size of 750 households at baseline and 750 at follow-up was obtained in the intervention (*N* = 1500) as well as the control area (*N* = 1500). This sample size was adequate to show a 10% effect size for stunting (primary outcome) among children aged less than 5 years at six years follow (40% in the control vs. 32.9% in the intervention) with more than 80% power and 5% significance level (two-sided test), a design effect of 2% and 5% sampling error. The sample size allowed for a 10% non-response rate. The sample size was adequately powered to detect a 6% effect size in the prevalence of wasting at six-year follow and to model associations between outcome and intervention, adjusted for demographics and other variables.

### 2.4. Evaluation Variables

The evaluation considered whether or not a district was exposure to the intervention. Outcome variables were anthropometric indices, namely height-for-age, weight-for-age, and weight-for-height. Weight and height data were collected by trained enumerators. Weight was measured using a SECA (Hamburg, Germany) digital scale to the nearest 0.1 kg. Height was measured using a measuring board made by Shorr Productions for use in survey settings to the nearest 0.1 cm. Children below two years of age were measured in supine position (lying down) while those over two years were measured standing up. Z scores for height-for-age (HAZ), weight-for-age (WAZ) and weight-height (WHZ) were generated using the 2006 World Health Organization (WHO) [36] and three types of child malnutrition were considered in this study: wasting (WHZ below minus two standard deviations and/or bilateral oedema), stunting (HAZ below minus two standard deviations), and underweight (WAZ below minus two standard deviations). To increase the accuracy of the anthropometric indices, implausible values were excluded. Biologically implausible values were defined using the WHO standards-based results as follows: z-scores of <−5 or >+5 for WHZ; <−6 or >+6 for HAZ; and <−6 or >+5 for WHZ [36].

The socioeconomic status as a potential confounding factor was measured using the household wealth index (HWI) and socio-demographic factors, with data collected using a structured questionnaire as per the Demographic and Health Survey’s (DHS’s) module [37]. The HWI was computed according to the DHS’s module [37] and was a composite measure of a household’s cumulative living standard. It was generated using the principal component analysis to produce the relative economic status of households based on an analysis ownership of selected assets, including televisions and bicycles; materials used for housing construction (e.g., the type of floor, wall, and roof materials); members per sleeping room; agricultural land (e.g., ownership of agricultural land and the amount of land owned); farm animals/livestock (e.g., ownership of farm animals and the numbers of different types of animals); and the types of water access and sanitation facilities (e.g., source of drinking water as well as the type of toilet and sharing of toilets). The socio-demographic factors included paternal and maternal literacy, paternal and maternal educational attainment, child age (from the child’s birth records/certificates), gender and caste or ethnicity. One question to account for the fluctuation in food availability was introduced as confounding factors: “Have you experienced food shortage in your household in the last one year?” on a yes/no response format.

### 2.5. Statistical Tests

Data were analysed using STATA version 14 (StataCorp, College Station, TX, USA). Descriptive statistics (e.g., means and frequencies) were used to summarise key variables. Propensity scores were used in difference-in-differences models to estimate the project impact. While propensity scores are an increasingly common matching method to improve covariate balance, there exist multiple matching methods with varying levels of model improvements associated with them [38,39,40]. However, authors often fail to report different models they assessed and tend to only summarise the best model that fits their data [38,39,40]. We move away from this practice and present three matching algorithms for our data: nearest-neighbour matching, kernel matching, and radius matching [39,40]. A logit model was used to estimate program participation (probability of being or not being in the intervention) as a function of household size, household wealth index, ethnicity, father’s education, child age, and child gender. We then used the predicted values from logit to generate propensity score for all households in the intervention and comparison group. The covariate balance was satisfactory. Finally, households in the intervention and comparison group were matched based on their similar propensity scores. The balance check was used to evaluate the effectiveness of the matching method. The standardized bias for each covariate in the propensity score model. We have both a continuous variable (e.g., child’s age in month) and categorical variables expressed as a set of binary indicators. The standardized bias was calculated by dividing the difference in means (continuous variables) or proportions (binary variables) of the covariate between the intervention and comparison group by the standard deviation [41]. Standardized biases of less than 25% were considered good balance between the groups [41].

**Figure 2:**
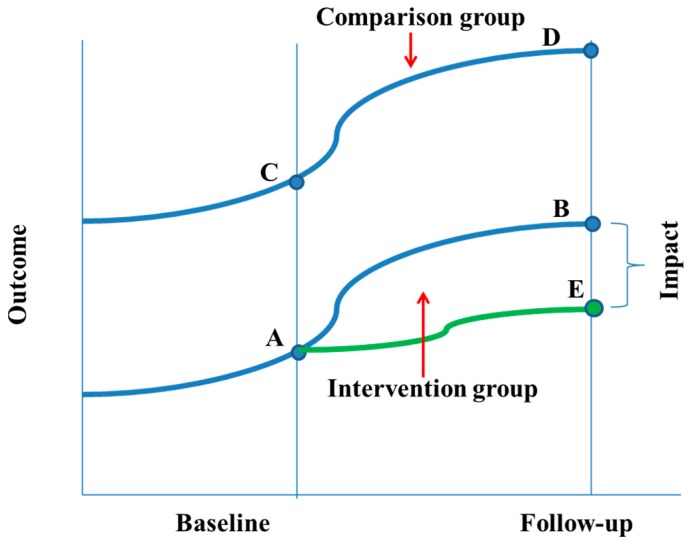
Difference in differences.

To detect the project impact, the difference-in-differences (DD) method was used to compare the changes over time between the intervention and comparison groups (Figure 2). The first approach involved estimating weighted DD coefficients of the unmatched sample with bootstrapping; accounting for clustering within wards and adjusted for household size, household wealth index, ethnicity, father’s education, child age, and child gender. Then propensity scores were used in DD models. Although matching on the propensity score is commonly used for removing the effects of confounding due to observed covariates [42], subjects with similar propensity scores tend to have the same distribution of measured baseline covariates; with matched intervention and comparison subjects having measured baseline covariates that are more likely to be similar to one another than are the baseline covariates of two unmatched subjects [43,44]. Consequently, matching on the propensity score has a high likelihood of inducing a within-matched pair correlation, leading to variance estimation [44,45]. The bootstrap has been recommended as one way to address variability of estimated program effect [44]. Therefore, bootstrap methods were used by drawing bootstrap samples from the matched pairs in the propensity-score-matched sample [44]. This method results in improved estimates of the standard error [44]. In all cases *p* < 0.05 was considered to be statistically significant.

## 3. Results

**Figure 3:**
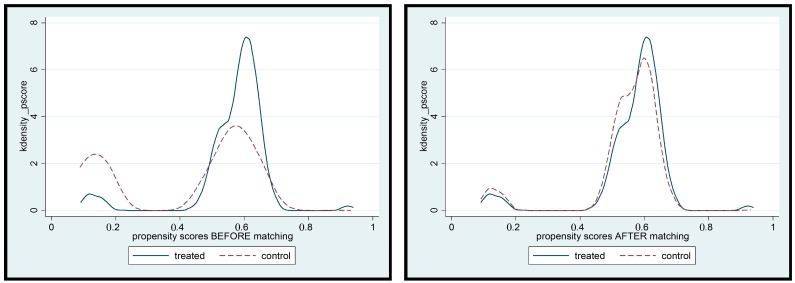
Assessing the adequacy of the matching approach.

Summary statistics of the matching variables and estimates of logit regression models for stage 1 of propensity score matching are summarised in Table 2. Estimates of standardized bias are reported in the last column of Table 3 and considerably much less than the 25% threshold recommended for balanced covariate. Figure 3 shows the empirical distribution of propensity scores before and after matching for households in the intervention and control group. In general, the empirical distributions of households in the intervention and comparison group track each other well after matching.

### Child Malnutrition

During the five-year study period, the prevalence of wasting increased slightly in the comparison group (from 5.8%; 95% CI: 4.3, 7.7% to 6.4%; 95% CI: 4.9, 8.4%, *p* = 0.593), although this increase was not statistically significant. However, the prevalence of underweight decreased significantly, from 37.3% (95% CI: 33.8, 40.8%) at baseline to 28.9% (95% CI: 25.8, 32.3%, *p* < 0.01). In contrast, the prevalence of wasting and underweight decreased significantly in the intervention group, from 12.7% (95% CI: 10.4, 15.2%) and 50.7% (95% CI: 47.1, 54.3%) at baseline to 5.7% (95% CI: 4.3, 7.6%) and 34.8% (95% CI: 31.5, 38.3%) at follow-up, respectively, at *p* < 0.001. Adjusted DD coefficients from the unmatched sample and those generated using propensity scores are summarised in Table 4. Overall, propensity score matched/weighted models produced better results than the unmatched analyses.

Our results suggest that the three matching estimators produced different effects on outcomes. The radius matching algorithm produced more robust results than the nearest neighbour or kernel matching estimators, and hence we report findings from the radius matching. The intervention had a positive impact on height-for-age z-scores (DD = 0.18; 95% CI: 0.09, 0.27, *p* < 0.05), weight-for-age z-scores (DD = 0.22, 95% CI: 0.15, 0.19, *p* < 0.01), and weight-for-height z-scores (DD = 0.19; 95% CI: 0.09, 0.30, *p* < 0.05).

The intervention resulted in a 5.16 (95% CI: 9.55, 0.77), 7.35 (95% CI: 11.62, 3.08) and 2.84 (95% CI: 5.58, 0.10) percentage point reduction in the proportion of children under the age of five who were stunted, underweight and wasted respectively. Among boys, the intervention resulted in a 6.15 (95% CI: 11.76, 0.53) and a 3.33 (95% CI: 6.16, 0.49) percentage point reduction in the prevalence of stunting and wasting respectively, but no impact was observed for underweight. Among girls, improvements were observed only for underweight, with a 9.02 (95% CI: 15.10, 2.94) percentage point reduction in the prevalence of underweight. No impact was observed for stunting or wasting. The analysis by children’s age groups revealed that the intervention resulted in a 6.66 (95% CI: 2.13, 3.18), 11.40 (95% CI:16.66, 6.13), and 4.10 (95% CI: 6.43, 1.78) percentage point reduction in the prevalence of stunting, underweight, and wasting among older children (≥24 months). No impact was observed among children younger than two years (Table 4; radius matching).

## 4. Discussion

This is the first ever study to examine the synergetic effect of targeted resource transfers for families, capacity building for social protection, and child cash grant on children’s nutritional status in Nepal. While social protection programs have been widely recognised as a key instrument in tackling child malnutrition, the evaluation of existing programs have focused on the conditionality (conditional vs. unconditional) of social protection programs, the duration and the amount of cash transfers, and targeting approaches and coverage (e.g., broad universal vs. targeted programs) with varied results [46]. The overall pattern suggests inconclusive evidence of a positive impact of cash transfer programs on child nutritional status and a lack of understanding of the pathways of impact [33,46,47].

Our findings that the intervention had an impact in child stunting, underweight, and wasting somehow mirror the literature. The added value of our study is the intervention’s impact on child stunting overall and among children aged two years or older. Our findings are from a radius matching algorithm and are not supported by findings from the nearest neighbour or kernel matching estimators. Caliendo and Kopeinig summarise the pros and cons of each approach [40]. In the nearest neighbour matching algorithm, a child from a household in the comparison group is chosen as a matching partner for a child from a household in the intervention group that is closest in terms of propensity score. However, the nearest neighbour matching faces the risk of bad matches when the closest neighbour is far away [40] Similarly, the kernel matching estimator uses weighted averages of all individuals in the comparison group to construct the counterfactual outcome and thus more information is used, leading to lower variance. Nevertheless, there is a high likelihood of observations being used that are bad matches [40]. Finally, the radius matching uses not only the nearest neighbour within each caliper, but all of the comparison members within the caliper [40]. By imposing a radius, matching quality rises as bad matches are avoided [40]. That is, within the imposed radius, the approach uses as many comparison units as are available, allowing for usage of more or fewer units when good matches are or are not available [40,48]. Our findings that the nearest neighbour matching produced poorer estimates than the original unmatched sample is consistent with emerging findings in this field [38].

Data from the radius matching estimators in our models are consistent with the literature. A number of systematic reviews and meta-analyses have been undertaken to evaluate the impact of cash transfers on child malnutrition. A systematic review by Bassett [33] examined whether conditional cash transfers reduce child malnutrition. The review found that cash transfers that were roughly less than 20% of household expenditure (<$US15) had no impact on stunting. Those with cash transfers approximating 20% or higher of the household expenditure reported a significant reduction in stunting, a reduction of six to 10 percentage points. However, the reduction in the prevalence of stunting was not uniformly distributed across the studies, with some studies reporting significant reductions among children aged two years or younger only and no impact among those older than two years. We did not find an intervention impact among children younger than two years. Nevertheless, some limited studies also reported reductions in stunting prevalence in children aged two years or older, which are consistent with our findings.

A meta-analysis by Manley et al. [47] evaluated how effective are cash transfer programs at improving child malnutrition using data from 18 programmes in 11 countries. They found that HAZ was the predominantly used anthropometric outcome, with some use of WAZ and WHZ. Overall, the pooled analysis found that cash transfer programs had no effect of child malnutrition. No difference was found between conditional and unconditional cash transfer programs. However, the effect of cash transfer programs on HAZ diverged greatly in their effectiveness, with higher marginal effects found in the most disadvantaged areas, among girls than boys, and in countries with poorer health care systems. Half of the cash transfer programs were found to have positive effects and another half found to have negative effects on WAZ and WHZ.

Recently, de Groot and colleagues [46] completed a review examining the impact of cash transfer programs on child malnutrition and sought to identify pathways of impact. They reported positive impacts on child nutritional outcomes in several countries. They also found several studies reporting either no significant impact or negative impact of cash transfer programs on child nutritional status. The authors documented a number of factors that may help explain some of the heterogeneous impacts of cash transfer programs on child nutrition. They concluded that factors that matter are the size of the transfer, the age of the child, targeting strategies, the access and quality of services, and the duration of program participation. They noted that larger transfer amounts are associated with greater probability of beneficiaries’ compliance with the conditions, but the overall pattern suggests inconclusive evidence of a positive impact on child nutritional status and a lack of understanding of the pathways of impact.

We found that the intervention had little impact among children younger than two years. It is possible that in our study a cash payment of NRs200 ($US2) per eligible family might not be large enough to affect infant and young child feeding practices. While studies examining the impact of cash transfers on the specific caregiver behaviours, the health of the caregiver as well as breastfeeding, diet diversity, and meal frequency are known to be associated with nutritional status of children younger than two years [46]. In their systematic review of the impact of cash transfers on the determinants of child nutrition, de Groot et al. [46] found that the impact varied. In some studies, cash transfer programs increased household expenditures, but not the caloric intake of children. In others, cash transfers increased children’s consumption of more nutritious food, high-protein food intake, and dietary diversity.

Several studies have found that the size of the transfer matters [30,46]. For example, a study looked at the impact of different cash-based intervention modalities on child and maternal nutritional status in Sindh Province, Pakistan, at six months and at one year using a cluster randomised controlled trial [30]. The intervention included a monthly unconditional cash transfer as either a standard cash payment of 1500 Pakistani rupees (PKR) or a double cash payment of 3000 PKR, a monthly fresh food voucher of 1500 PKR; and a control group that received no specific intervention. It found a significant reduction in child wasting in the double cash arm after six months when compared to the control group. Significant improvements in weight-for-height were also observed in both the fresh food voucher and double cash arms. All three intervention groups showed similar significantly lower odds of being stunted at six months than the control group. The study concluded that the amount of cash given had the greatest effect on wasting but only at six months, and impacts after six months were only seen for stunting regardless of the intervention modality [30]. The intervention effect on stunting was unexpected findings given that stunting is a chronic condition and the short-term nature of the interventions. Our study was implemented over five years, hence the robustness of our findings on stunting.

While the success of cash transfers programs has heavily relied on targeting the poor and at-risk populations have [33,34,46,47,49], the effectiveness of targeting approaches is affected by socio-political and economic factors including the weak and uncoordinated targeting of the poor, the high cost of delivering such a program, and delays in disbursements. The strengths of our intervention were its multi-sectoral approach and coordination, the strong focus on child sensitive social protection program augmented with capacity building for effective social protection. The effectiveness of targeted cash transfer program is greater when implemented in settings where utilisation of nutrition interventions is low, but the intervention is linked to nutrition-promoting activities among at risk populations [33], a strategy which was strongly embedded in our intervention approach and could explain the greater impact on wasting and underweight.

Finally, our child sensitive social protection program and capacity building for effective social protection included nutritional education campaigns embedded within existing universal social transfer programs, hence increasing their social acceptability and political appeal. Such an approach facilitates the adoption of nutritionally-oriented programs that are more inclusive and have high reach.

There are a number of limitations and strengths worth outlining. The lack of random assignment into intervention groups means that the comparison and intervention groups were non-equivalent, which threatened both the external and internal validity of our findings. However, this was addressed by the propensity matching scores. Our design sought to maximise the trade-off between experimental control and ecological validity. Given that randomisation was impractical, the quasi-experimental design was the most appropriate design and provided adequate avenues for testing the effectiveness of community-based interventions in real-world settings [35]. Because the intervention did not involve random assignment, its acceptability to the broader society was high. However, there was a high risk of contamination, requiring the need to have a “buffer” zone. The comparison community (Bajhang District) is in Seti Zone in the Far-Western Development Region. In contrast, the intervention community (Kalikot District) is in Karnali Zone in the Mid-Western Development Region. The two districts were chosen because of their similar socio-demographic, economic, and child malnutrition profile, but the distance between them acted as a buffer zone hence minimising the risk of contamination. The intervention was embedded within existing universal social transfer programs hence ensuring continuity of participation and preventing the disruption in disbursements. The implementation of the intervention involved too many stakeholders with differing expectations and competing objectives, which might have hampered the effective implementation of the project. This challenge was overcome by having clear role and responsibilities and a focal coordinating committee overseen by the GoN.

## 5. Conclusions

Notwithstanding the above limitations, our study is the first to our knowledge to evaluate child-sensitive social protection program, augmented by capacity building for effective social protection and embedded within existing universal social transfer programs. Our results suggest that such an approach can address child wasting and underweight effectively. Based on available evidence, in order to increase the intervention effectiveness on younger children, the child cash payment amount needs to be revisited and closely embedded into infant and young child feeding initiatives. It also needs to be adjusted to equate to 20% of household expenditure or more to maximize the diversity of food available to young children.

## Figures and Tables

**Table 1:** UNICEF-supported intervention to improve child health outcomes through CCG (child cash grant) programs.

Program Activities	Program Activities in the Intervention Area (5 Districts of the Karnali Zone)	Program Activities in the Control Area (Bajhang Distrcict)	No. of Activities per Year	Frequency of Delivery	Mode of Delivery
Advocacy and Capacity Building					
Program planning and review workshop	Yes	No	Planning undertaken Once every year, review workshop done for twice (during 2013 and 2015)	3 times in each districts during the project period—Review twice (during mid-implementation and final review)	Planning Meeting Workshop
Training in Infant and Young Child Feeding (IYCF) to all health workers at district level	Yes	No	In 2011 (Starting year) and Refresher in 2015	One IYCF ToT (Training of Trainer) and Cascading initially, review were taken annually and refresher training in 2015	ToT jointly by Program Cooperation Agreement (PCA) partner as well as Ministry of Health (MoH), Refresher undertaken by MoH mechanisms
Training to all health workers at health facility (VDC) level	Yes	No	In 2011 (Starting year) and Refresher in 2015	One IYCF TOT and Cascading initially, review were taken annually and refresher training in 2015	ToT jointly by PCA partner as well as MoH, Refresher undertaken by MoH mechanisms
Training to Female Community Heath Volunteers (FCHV), Traditional Healers, teachers and community leaders	Yes	No	In 2011 (Starting year) and Refresher in 2015	twice	training/orientation Implementation through FCHV (Government/MoH) mechanisms, facilitated by Non Government Organization (NGO) partners
Facilitate Mothers Group Meeting on Nutrition at community level (at least one in each ward)	Yes	No	Mothers Group meeting happens every month in each settlement through Female Community Heath Volunteers	12 times/ year/FCHV in all districts	Through MoH mechanisms
Orientation to social mobilizers and community facilitators in all five districts of Karnali.	Yes	No	One a year (in 2012 and 2015)	twice	orientation through NGO partner
Conduct 1 day VDC level refresher training/program orientation to FCHV in each VDC in Karnali zone (Ensure IYCF-Cash Grant refresher conducted by Heath post/District Public Health Offices)	Yes	No	One training and One Refresher in 5 years	twice	Through MoH mechanisms
Awareness raising					
Orientation/meeting to mother/caregivers in all 5 districts (134 VDCs) of Karnali and demonstration of preparation of complementary food lito (mixtures of roasted and grinded cereals, legumes) and jaulo (cooked rice and lentils with vegetables and ghee) for children	Yes	No	twice in 5 years	2	Implementation through FCHVs (Government/MoH) mechanisms, facilitated by PCA partners (NGO)
Dissemination of Behaviour Change Communication message during orientations at 5 districts (134 VDCs) of Karnali	Yes	No	1	5	Implementation through Government/MoH (Health facilities, Health workers, FCHVs, etc.) mechanisms, facilitated by PCA partners (NGO)
Production of radio program on nutrition and social protection promotion.	Yes	No	1	5	Through Local Frequency Modulation (FM) Radio stations facilitated by NGO partners
Broadcasting the Nutrition and Social Protection related Radio Programmes through FM Radios	Yes	No	1	5	Through Local FM stations facilitated by NGO partners
Conduct local food preparation and demo at mother group/school and Early Childhood Development (ECD)	Yes	No	1	5 (Once every year)	Through Government/MoH (Health workers, FCHVs, etc.) mechanisms at community level
Partnership with community Radios for producing and broadcasting the weekly magazine on child feeding, caring, nutrition, hygiene and sanitation.	Yes	No	1	365 days	Through Local FM stations facilitated by NGO partners
Daily Airing of Jingle Message from FM radios.	Yes	No	1	365 days	Through Local FM stations facilitated by NGO partners
Promote nutritious food (eggs, Jaulo) for underweight children and iodized salt to all children 0–23 coming to Growth Monitoring and pregnant women coming to Ante-Natal Checkups	Yes	No	1 time in five year	1	Implementation through Government/MoH (Health facilities, Health workers, FCHVs, etc.) mechanisms, facilitated by NGO partners
Mobilization of other community structures like women’s group					
Conduct wards level discussion/meeting through community structure	Yes	No	1	5	Through Government/MoH mechanisms, facilitated by NGO partners
Conduct joint monitoring and Evaluation at VDC level from District Resource Group and NGO Partner	Yes	No	1	2	Through Government/MoH mechanisms, facilitated by NGO partners
Community Facilitator for mobilization of community groups/institutions (2 persons in each district for 5 months)	Yes	No	1	3 times (2011, 2012, 2015)	Through Government/MoH mechanisms, facilitated by NGO partners
Community Level Dramas at cluster level in all 5 district of Karnali	Yes	No	1	2 times in each clusters	Through Government/MoH mechanisms, facilitated by NGO partners
Distribution of Child Grant to all under 5 children (in every four months) by the Government of Nepal	Yes	Yes (only to Dalit Children—10% of total under five children)	3 times per year	3 times per year in all 5 districts every year	Through Local government (VDCs)
Birth Registration Campaign					
Conduct VDC level birth registration campaign promoting birth registration within 35 days in all 134 VDCs of Karnali	Yes	No	1	2 (in 2012 and 2015) in all 5 districts	Through Local Government at district and village levels (District Development Committee (and Village Development Committees), facilitated by NGO partners
Provide incentive for those families who do birth registration within 35 days of child birth	Yes	No	1	1 times in all 5 districts	Through Local Government at district and village levels, facilitated by NGO partners
Community Facilitator for mobilization of community groups/institutions(2 persons in each district for 5 months)	Yes	No	1	3 times (2011, 2012, 2015)	Through Government/MoH mechanisms, facilitated by NGO partners
System Strengthening to Management Information System (MIS) of Social Protection Schemes (Child Grant, Old age Pension, Single Women Allowances, etc.)					
Provide technical support to DDC and KIRDARC for Information Technology support and MIS rollout in all five districts of Karnali through consultants.	Yes	No	1	1 times in each districts	
Community Facilitator for mobilization of community groups/institutions(2 persons in each district for 5 months)	Yes	No	1	3 times (2011, 2012, 2015)	Through Government/MoH mechanisms, facilitated by NGO partners
Supervision and Monitoring including process Monitoring	Yes	No	Ongoing	ongoing	Through Government/MoH mechanisms

**Table 2:** Summary statistics of the matching variables and estimates of logit regression models for stage 1 of propensity score matching.

Matching Variables	All			Intervention						Control						Logit Model		
				Baseline			Follow-Up			Baseline			Follow-Up					
	*N*	Mean	SD	*N*	Mean	SD	*N*	Mean	SD	*N*	Mean	SD	*N*	Mean	SD	Coefficient	SE	*p*-Value
People per household	3000			750			750			750			750					
4 people or less		15.3%	36.0%		13.5%	34.2%		21.2%	40.9%		15.3%	36.1%		15.3%	36.1%	0.40	0.14	0.004
5–8 people		63.8%	48.1%		64.8%	47.8%		65.2%	47.7%		60.5%	48.9%		60.5%	48.9%	0.16	0.10	0.111
9 people or above		20.8%	40.6%		21.7%	41.3%		13.6%	34.3%		24.1%	42.8%		24.1%	42.8%	**Ref**		
Household wealth index	2899			724			710			731			731					
Poor		60.0%	49.0%		89.1%	31.2%		54.2%	49.9%		10.1%	30.2%		10.1%	30.2%	2.17	0.13	0.000
Middle class		20.0%	40.0%		9.7%	29.6%		35.9%	48.0%		23.9%	42.7%		23.9%	42.7%	2.08	0.15	0.000
Rich		20.0%	40.0%		1.2%	11.1%		9.9%	29.8%		65.9%	47.4%		65.9%	47.4%	**Reference**		
Child’s age in months	3000	27.98	15.53	750	28.66	15.36	750	28.4	15.71	750	28.08	15.55	750	28.08	1555.0%	0.01	0.00	0.045
Child’s gender	3000			750			750			750			750					
Girl		43.4%	49.6%		44.8%	49.8%		43.6%	49.6%		43.7%	49.6%		43.7%	49.6%	Reference		
Boy		56.6%	49.6%		55.2%	49.8%		56.4%	49.6%		56.3%	49.6%		56.3%	49.6%	−0.08	0.08	0.322
Ethnicity	3000			750			750			750			750					
Disadvantage ethnic groups		0.4%	6.6%		1.5%	12.0%		0.1%	3.7%		0.0%	0.0%		0.0%	0.0%	2.04	1.04	0.050
Dalit Hill/Terai		21.1%	40.8%		21.3%	41.0%		25.5%	43.6%		16.8%	37.4%		16.8%	37.4%	0.01	0.10	0.911
Upper caste Group		78.5%	41.1%		77.2%	42.0%		74.4%	43.7%		83.2%	37.4%		83.2%	37.4%	Reference		
Father’s education	3000			750			750			750			750					
Primary or less		12.6%	33.2%		2.1%	14.5%		16.8%	37.4%		25.9%	43.8%		25.9%	43.8%	0.27	0.14	0.052
Secondary level		30.0%	45.8%		33.1%	47.1%		22.3%	41.6%		26.4%	44.1%		26.4%	44.1%	−0.05	0.14	0.744
Intermediate or higher		57.4%	49.5%		64.8%	47.8%		60.9%	48.8%		47.7%	50.0%		47.7%	50.0%	Reference		

**Table 3:** Evaluation of standardized differences in matched sample.

	Intervention		Comparison		%Bias
	Unmatched	Matched	Unmatched	Matched	
**No. of people per household**					
4 people or less	0.159	0.082	0.128	0.118	−10.30
5–8 people	0.657	0.664	0.629	0.659	1.20
9 people or more	0.184	0.254	0.243	0.223	7.40
**Household wealth index**					
Poor	0.717	0.648	0.484	0.683	−7.40
Middle class	0.227	0.275	0.174	0.240	8.80
Rich	0.056	0.077	0.342	0.077	0.00
**Child’s age in months**	28.341	25.429	27.476	27.602	−14.00
**Child’s gender**					
Girl	0.438	0.395	0.429	0.421	5.30
Boy	0.562	0.605	0.571	0.579	−5.30
**Ethnicity**					
Disadvantage ethnic groups	0.008	0.001	0.001	0.001	0.00
Dalit Hill/ Terai	0.224	0.208	0.179	0.212	−1.00
Upper caste Group	0.768	0.791	0.820	0.787	1.00
**Father’s education**					
Primary or less	0.630	0.496	0.519	0.540	−8.90
Secondary level	0.277	0.378	0.323	0.338	8.70
Intermediate or higher	0.438	0.395	0.429	0.421	−5.30

**Table 4:** Program impact on child undernutrition.

	Original Dataset							Matched Dataset: Matching Algorithms								
	Comparison	Intervention	Comparison	Intervention				Kernel !			Nearest Neighbour !			Radius !#		
	*N* = 748	*N* = 743	*N* = 749	*N* = 750	ADD	95% CI		ADD	95% CI		ADD	95% CI		ADD	95% CI	
**Girls ^a^**																
Height	77.2 (10.3)	77.8 (10.9)	78.7 (11.1)	78.8 (11.7)	0.17	−0.05	0.40	0.65	−0.87	2.18	0.01	−1.43	1.45	0.69	−0.99	2.36
Weight	9.3 (2.4)	9.3 (2.5)	9.7 (2.6)	9.8 (2.9)	0.31 ***	0.22	0.40	0.32	−0.06	0.71	0.13	−0.25	0.51	0.33 *	0.06	0.6
HAZ	−2.3 (1.3)	−2.6 (1.4)	−2.1 (1.3)	−2.2 (1.3)	0.21	−0.01	0.44	0.11	−0.06	0.27	0.07	−0.18	0.32	0.15	−0.06	0.36
WAZ	−1.7 (1.0)	−2.1 (1.1)	−1.5 (1.1)	−1.6 (1.1)	0.33 ***	0.23	0.44	0.17 *	0.06	0.28	0.13	−0.1	0.37	0.19 *	0.09	0.29
WHZ	−0.5 (0.9)	−0.8 (1.1)	−0.5 (1.0)	−0.4 (1.0)	0.31 ***	0.15	0.46	0.17 *	0.05	0.3	0.13	−0.06	0.33	0.18	−0.01	0.36
Stunting	61.9	68	55.5	61	−3.98	−15.44	7.48	−2.65	−9.15	3.85	−5.07	−11.78	1.63	−4.24	−10.4	1.93
Underweight	37.1	53.1	30.8	34.9	−16.25 ***	−24.12	−8.38	−7.83 ***	−14.39	−1.26	−8.89	−18.96	1.17	−9.02 ***	−15.1	−2.94
Wasting	4.5	9.3	7	4.9	−9.29 ***	−15.86	−2.72	−2.62	−6.33	1.09	−3.31	−8.2	1.58	−2.47	−5.9	0.95
**Boys ^a^**																
Height	80.2 (11.2)	80.6 (11.2)	82.4 (11.2)	81.6 (11.8)	−0.05	−1.17	1.06	0.21	−1.31	1.74	0.13	−1.13	1.39	0.22	−0.9	1.35
Weight	10.2 (2.6)	10.2 (2.7)	10.9 (2.8)	10.7 (3.0)	0.17	−0.17	0.52	0.23	−0.11	0.57	0.21	−0.23	0.66	0.25	−0.09	0.6
HAZ	−2.4 (1.3)	−2.6 (1.5)	−2.0 (1.3)	−2.2 (1.4)	0.14	−0.14	0.43	0.16 *	0	0.31	0.08	−0.17	0.33	0.22 *	0.08	0.35
WAZ	−1.7 (1.0)	−2.1 (1.1)	−1.4 (1.1)	−1.6 (1.1)	0.26	0.01	0.51	0.19 **	0.1	0.29	0.17 *	0.01	0.32	0.25 *	0.08	0.42
WHZ	−0.6 (0.9)	−0.9 (1.2)	−0.3 (1.1)	−0.4 (1.0)	0.27 ***	0.08	0.47	0.21 *	0.06	0.36	0.20 *	0.02	0.38	0.21 *	0.07	0.36
Stunting	63.7	65.7	50.8	58.8	0.69	−14.00	15.37	−4.14	−10.48	2.19	−1.27	−10.49	7.95	−6.15 *	−11.76	−0.53
Underweight	37.4	48.8	27.5	34.8	−9.74	−23.38	3.90	−5.03	−11.19	1.13	−3.39	−13.45	6.67	−6.49	−13.15	0.16
Wasting	6.6	15.3 ***	5.9	6.4	−9.55 ***	−14.46	−4.64	−3.11	−6.4	0.19	−3.54	−8.31	1.23	−3.33 *	−6.16	−0.49
**<2 years ^b^**																
Height	70.0 (6.5)	69.6 (6.7)	70.8 (7.4)	69.2 (7.4)	−0.28	−1.16	0.60	−0.85 *	−1.67	−0.02	−0.91	−2.45	0.63	−0.81 *	−1.6	−0.02
Weight	7.8 (1.5)	7.5 (1.6)	8.1 (1.8)	7.6 (1.8)	0.03	−0.30	0.37	−0.15	−0.38	0.08	−0.17	−0.45	0.11	−0.14	−0.36	0.08
HAZ	−2.0 (1.4)	−2.2 (1.5)	−1.6 (1.4)	−1.9 (1.5)	0.03	−0.21	0.28	0.12	−0.09	0.33	−0.1	−0.37	0.18	0.13	−0.08	0.33
WAZ	−1.5 (1.1)	−2.0 (1.2)	−1.2 (1.2)	−1.6 (1.2)	0.18	−0.04	0.41	0.08	−0.06	0.22	−0.01	−0.24	0.23	0.09	−0.08	0.27
WHZ	−0.6 (0.9)	−1.1 (1.3)	−0.5 (1.1)	−0.7 (1.1)	0.18	−0.04	0.41	0.05	−0.09	0.2	0.1	−0.15	0.34	0.07	−0.08	0.21
Stunting	52	58.2	39.8	50.8	2.76	−5.16	10.68	−2.48	−8.1	3.14	1.61	−6.44	9.66	−3.57	−10.37	3.23
Underweight	32.6	47.1	23.8	37.1	−5.39	−18.43	7.66	−0.46	−7.8	6.89	1.86	−8.42	12.15	−1.24	−8.08	5.6
Wasting	6.7	18.8	6.8	10.3	−9.19 ***	−15.81	−2.57	−1.2	−5.16	2.76	−1.91	−6.88	3.05	−1.03	−4.2	2.13
**≥2 years ^b^**																
Height	87.1 (7.1)	86.4 (7.9)	88.3 (7.1)	87.9 (7.6)	0.53	−0.12	1.18	0.41	−0.18	1.01	0.59	−0.45	1.63	0.74	−0.16	1.64
Weight	11.7 (1.9)	11.4 (2.0)	12.1 (2.0)	12.1 (2.1)	0.39 ***	0.12	0.66	0.36 ***	0.12	0.6	0.44 **	0.18	0.69	0.44 ***	0.25	0.63
HAZ	−2.6 (1.1)	−2.8 (1.2)	−2.4 (1.1)	−2.4 (1.3)	0.15	−0.02	0.31	0.17 *	0.06	0.28	0.12	−0.03	0.28	0.21 *	0.06	0.35
WAZ	−1.9 (1.0)	−2.1 (1.1)	−1.6 (1.0)	−1.6 (1.0)	0.28 ***	0.12	0.44	0.28 ***	0.18	0.37	0.27 **	0.13	0.41	0.30 ***	0.19	0.41
WHZ	−0.5 (0.9)	−0.6 (1.0)	−0.3 (1.0)	−0.2 (0.9)	0.29 ***	0.11	0.47	0.26 ***	0.17	0.35	0.29 **	0.12	0.46	0.27 ***	0.14	0.4
Stunting	73	73.1	62.8	65.8	0.05	−6.01	6.11	−4.82	−10.23	0.6	−4.05	−12.54	4.44	−6.66 **	−12.13	−1.18
Underweight	41.5	53.3	32.8	33.3	−14.87 ***	−23.27	−6.46	−10.45 ***	−16.02	−4.88	−9.2	−18.52	0.11	−11.40 ***	−16.66	−6.13
Wasting	4.9	8.2	6.1	2.7	−8.51 ***	−13.91	−3.11	−3.86 **	−5.98	−1.74	−6.22 **	−9.22	−3.22	−4.10 **	−6.43	−1.78
**All ^c^**																
Height	78.9 (10.9)	79.3 (11.1)	80.8 (11.3)	80.3 (11.9)	0.11	−0.51	0.72	0.42	−0.68	1.52	−0.11	−1.08	0.86	0.48	−0.33	1.28
Weight	9.8 (2.6)	9.8 (2.7)	10.4 (2.8)	10.3 (3.0)	0.26 **	0.05	0.47	0.27 *	0	0.55	0.17	−0.12	0.47	0.29	−0.01	0.6
HAZ	−2.3 (1.3)	−2.6 (1.4)	−2.1 (1.3)	−2.2 (1.4)	0.17 *	0.03	0.31	0.14 *	0.03	0.25	0.05	−0.12	0.23	0.18 *	0.09	0.27
WAZ	−1.7 (1.0)	−2.1 (1.1)	−1.4 (1.1)	−1.6 (1.1)	0.29 ***	0.15	0.44	0.19 **	0.11	0.28	0.18 *	0.07	0.29	0.22 **	0.15	0.29
WHZ	−0.5 (0.9)	−0.8 (1.1)	−0.4 (1.1)	−0.4 (1.0)	0.29 ***	0.15	0.42	0.18 *	0.09	0.28	0.24 *	0.08	0.4	0.19 *	0.09	0.3
Stunting	63	66.7	52.9	59.8	−1.34	−7.12	4.44	−3.51	−7.83	0.82	−2.18	−10.22	5.87	−5.16 *	−9.55	−0.77
Underweight	37.3	50.7	28.9	34.8	−12.54 ***	−19.82	−5.25	−6.29 ***	−10.96	−1.62	−5.19	−10.75	0.37	−7.35 ***	−11.62	−3.08
Wasting	5.8	12.7	6.4	5.7	−9.32 ***	−14.86	−3.79	−2.86 *	−4.91	−0.8	−4.84 ***	−8.62	−1.06	−2.84 **	−5.58	−0.1

* *p* < 0.05; ** *p* < 0.01; *** *p* < 0.001. ADD = Adjusted difference-in-differences. ^a^ Adjusted for father’s educational attainment, household wealth index, child age, caste/ethnicity, and family size; weighted with bootstrapping; ^b^ Adjusted for father’s educational attainment, household wealth index, caste/ethnicity, gender, and family size, weighted with bootstrapping; ^c^ Adjusted for father’s educational attainment, household wealth index, caste/ethnicity, gender, child age in month, and family size, weighted with bootstrapping. # Radius = 0.02; ! Weighted with bootstrapping. Z scores for height-for-age (HAZ), weight-for-age (WAZ) and weight-height (WHZ).
