# Correction: Renzaho, A.M.N., et al. The Synergetic Effect of Targeted Resource Transfers for Families, Child Sensitive Social Protection Programs, and Capacity Building for Effective Social Protection on Children’s Nutritional Status in Nepal. *Int. J. Environ. Res. Public Health* 2017, *14*, 1502

**DOI:** 10.3390/ijerph15050869

**Published:** 2018-04-26

**Authors:** Andre M. N. Renzaho, Stanley Chitekwe, Wen Chen, Sanjay Rijal, Thakur Dhakal, Pradiumna Dahal

**Affiliations:** 1 Humanitarian and Development Studies, School of Social Sciences and Psychology, Western Sydney University, Locked Bag 1797, Penrith NSW 2751, Australia; Wen.Chen@westernsydney.edu.au; 2 School of Public Health and Preventive Medicine, Monash University, The Alfred Centre, 99 Commercial Road, Melbourne VIC 3004, Australia; 3 UNICEF Nepal, Leknath Marg, Kathmandu 44600, Nepal; schitekwe@unicef.org (S.C.); sarijal@unicef.org (S.R.); tdhakal@unicef.org (T.D.); pdahal@unicef.org (P.D.); 4 Faculty of Medical Statistics and Epidemiology, School of Public Health, Sun Yat-sen University, Guangzhou 510080, China

The authors wish to add the following corrections to their paper published in the International Journal of Environmental Research and Public Health [1]. During the galley proof process, the production of the paper omitted the minus sign for the 95% CI of the results section on the project’s impact on child underweight, wasting, and stunting in the abstract (p. 1) and the manuscript (p. 15).

In the abstract, the sentence regarding the result should be:

“Propensity score matched/weighted models produced better results than the unmatched analyses, and hence we report findings from the radius matching. The intervention resulted in a 5.2 (adjusted difference-in-difference [ADID] = −5.16; 95% CI: −9.55, −0.77), 7.4 (ADID: −7.35; 95% CI: −11.62, −3.08) and 2.8 (ADID = −2.84; 95% CI: −5.58, −0.10) percentage point reduction in the prevalence of stunting, underweight, and wasting among children under the age of five, respectively. The intervention impact was greater in boys than girls for stunting and wasting; and greater in girls than boys for underweight. The intervention also resulted in a 6.7 (ADID = −6.66; 95% CI: −12.13, −1.18), 11.4 (ADID = −11.4; 95% CI: −16.66, −6.13), and 4.1 (ADID = −4.10; 95% CI: −6.43, −1.78) percentage point reduction in the prevalence of stunting, underweight, and wasting among older children (≥24 months). No impact was observed among younger children (<24 months).”

The last two paragraphs in page 15 should be:

Our results suggest that the three matching estimators produced different effects on outcomes. The radius matching algorithm produced more robust results than the nearest neighbor or kernel matching estimators, and hence we report findings from the radius matching. The intervention had a positive impact on height-for-age z-scores (adjusted difference-in-difference (ADID) = 0.18; 95% CI: 0.09, 0.27, *p* < 0.05), weight-for-age z-scores (ADID = 0.22, 95% CI: 0.15, 0.19, *p* < 0.01), and weight-for-height z-scores (ADID = 0.19; 95% CI: 0.09, 0.30, *p* < 0.05). 

The intervention resulted in a 5.2 (ADID = −5.16; 95% CI: −9.55, −0.77), 7.4 (ADID: −7.35; 95% CI: −11.62, −3.08) and 2.8 (ADID = −2.84; 95% CI: −5.58, −0.10) percentage point reduction in the proportion of children under the age of five who were stunted, underweight and wasted respectively. Among boys, the intervention resulted in a 6.2 (ADID = −6.15; 95% CI: −11.76, −0.53) and 3.3 (ADID = −3.33; 95% CI: −6.16, −0.49) percentage point reduction in the prevalence of stunting and wasting respectively, but no impact was observed for underweight. Among girls, improvements were observed only for underweight, with a 9.0 (ADID = −9.02; 95% CI: −15.10, −2.94) percentage point reduction in the prevalence of underweight. No impact was observed for stunting or wasting. The analysis by children’s age groups revealed that the intervention resulted in a 6.7 (ADID = −6.66; 95% CI: −12.13, −1.18), 11.4 (ADID = −11.40; 95% CI: −16.66, −6.13), and 4.1 (ADID = −4.10; 95% CI: −6.43, −1.78) percentage point reduction in the prevalence of stunting, underweight, and wasting among older children (≥24 months). No impact was observed among children younger than two years (Table 4; radius matching).

We deleted the word “baseline” in Figure 1:

**Figure 1:**
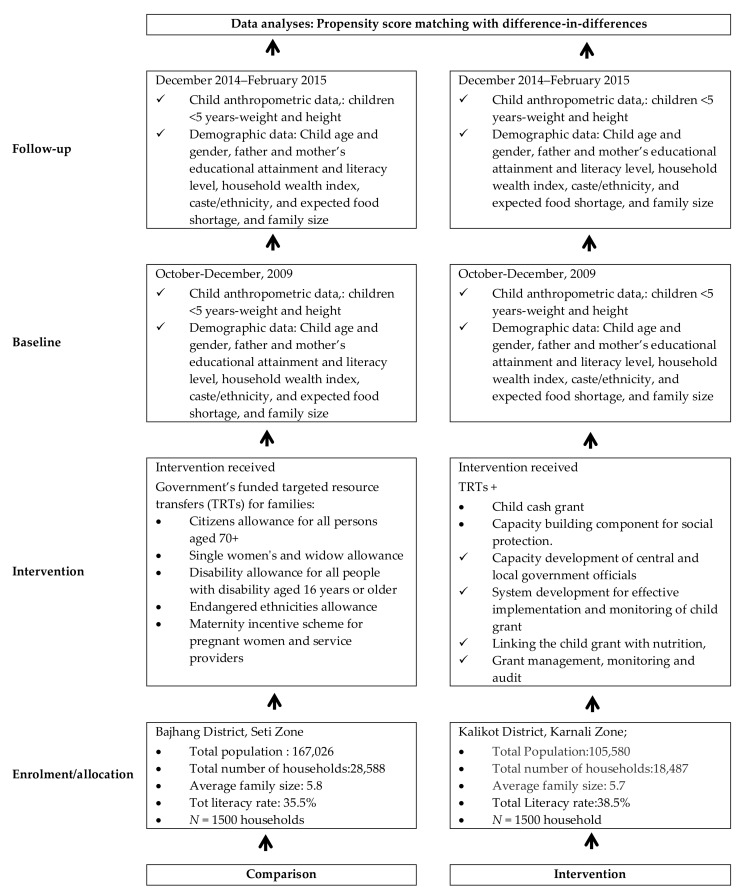
Flow diagram detailing the intervention implementation plan and data collection phases.

We also made some changes on Tables 2–4; therefore, the Tables should be as follows:

**Table 2:** Summary statistics of the matching variables and estimates of logit regression models for stage 1 of propensity score matching.

Matching Variables	All			Intervention						Control						Logit Model		
				Baseline			Follow-Up			Baseline			Follow-Up					
	*N*	Mean	SD	*N*	Mean	SD	*N*	Mean	SD	*N*	Mean	SD	*N*	Mean	SD	Coefficient	SE	*p*-Value
People per household	3000			750			750			750			750					
4 people or less		15.3%	36.0%		13.5%	34.2%		21.2%	40.9%		15.3%	36.1%		15.3%	36.1%	0.40	0.14	0.004
5–8 people		63.8%	48.1%		64.8%	47.8%		65.2%	47.7%		60.5%	48.9%		60.5%	48.9%	0.16	0.10	0.111
9 people or above		20.8%	40.6%		21.7%	41.3%		13.6%	34.3%		24.1%	42.8%		24.1%	42.8%	**Ref**		
Household wealth index	2899			724			710			731			731					
Poor		60.0%	49.0%		89.1%	31.2%		54.2%	49.9%		10.1%	30.2%		10.1%	30.2%	2.17	0.13	0.000
Middle class		20.0%	40.0%		9.7%	29.6%		35.9%	48.0%		23.9%	42.7%		23.9%	42.7%	2.08	0.15	0.000
Rich		20.0%	40.0%		1.2%	11.1%		9.9%	29.8%		65.9%	47.4%		65.9%	47.4%	**Reference**		
Child’s age in months	3000	27.98	15.53	750	28.66	15.36	750	28.4	15.71	750	28.08	15.4	750	28.08	15.4	0.01	0.00	0.045
Child’s gender	3000			750			750			750			750					
Girl		43.4%	49.6%		44.8%	49.8%		43.6%	49.6%		43.7%	49.6%		43.7%	49.6%	Reference		
Boy		56.6%	49.6%		55.2%	49.8%		56.4%	49.6%		56.3%	49.6%		56.3%	49.6%	−0.08	0.08	0.322
Ethnicity	3000			750			750			750			750					
Disadvantage ethnic groups		0.4%	6.6%		1.5%	12.0%		0.1%	3.7%		0.0%	0.0%		0.0%	0.0%	2.04	1.04	0.050
Dalit Hill/Terai		21.1%	40.8%		21.3%	41.0%		25.5%	43.6%		16.8%	37.4%		16.8%	37.4%	0.01	0.10	0.911
Upper caste Group		78.5%	41.1%		77.2%	42.0%		74.4%	43.7%		83.2%	37.4%		83.2%	37.4%	Reference		
Father’s education	3000			750			750			750			750					
Intermediate or higher		12.6%	33.2%		2.1%	14.5%		16.8%	37.4%		5.6%	23.1%		25.9%	43.8%	Reference		
Secondary level		30.0%	45.8%		33.1%	47.1%		22.3%	41.6%		38.3%	48.6%		26.4%	44.1%	−0.05	0.14	0.744
Primary or less		57.4%	49.5%		64.8%	47.8%		60.9%	48.8%		56.1%	49.7%		47.7%	50.0%	0.27	0.14	0.052

**Table 3:** Evaluation of standardized differences in matched sample.

	Intervention		Comparison		% Bias
	Unmatched	Matched	Unmatched	Matched	
**No. of people per household**					
4 people or less	0.159	0.082	0.128	0.118	−10.30
5–8 people	0.657	0.664	0.629	0.659	1.20
9 people or more	0.184	0.254	0.243	0.223	7.40
**Household wealth index**					
Poor	0.717	0.648	0.484	0.683	−7.40
Middle class	0.227	0.275	0.174	0.240	8.80
Rich	0.056	0.077	0.342	0.077	0.00
**Child’s age in months**	28.341	25.429	27.476	27.602	−14.00
**Child’s gender**					
Girl	0.438	0.395	0.429	0.421	5.30
Boy	0.562	0.605	0.571	0.579	−5.30
**Ethnicity**					
Disadvantage ethnic groups	0.008	0.001	0.001	0.001	0.00
Dalit Hill/Terai	0.224	0.208	0.179	0.212	−1.00
Upper caste Group	0.768	0.791	0.820	0.787	1.00
**Father’s education**					
Primary or less	0.630	0.496	0.519	0.540	−8.90
Secondary level	0.277	0.378	0.323	0.338	8.70
Intermediate or higher	0.093	0.127	0.158	0.122	−1.20

**Table 4:** Program impact on child undernutrition.

	Original Dataset							Matched Dataset: Matching Algorithms								
	Comparison	Intervention	Comparison	Intervention				Kernel !			Nearest Neighbor !			Radius !#		
	*N* = 748	*N* = 743	*N* = 749	*N* = 750	ADID	95% CI		ADID	95% CI		ADID	95% CI		ADID	95% CI	
Girls ^a^																
Height	77.2 (10.3)	77.8 (10.9)	78.7 (11.1)	78.8 (11.7)	0.17	−0.05	0.40	0.65	−0.87	2.18	0.01	−1.43	1.45	0.69	−0.99	2.36
Weight	9.3 (2.4)	9.3 (2.5)	9.7 (2.6)	9.8 (2.9)	0.31 ***	0.22	0.40	0.32	−0.06	0.71	0.13	−0.25	0.51	0.33 *	0.06	0.6
HAZ	−2.3 (1.3)	−2.6 (1.4)	−2.1 (1.3)	−2.2 (1.3)	0.21	−0.01	0.44	0.11	−0.06	0.27	0.07	−0.18	0.32	0.15	−0.06	0.36
WAZ	−1.7 (1.0)	−2.1 (1.1)	−1.5 (1.1)	−1.6 (1.1)	0.33 ***	0.23	0.44	0.17 *	0.06	0.28	0.13	−0.1	0.37	0.19 *	0.09	0.29
WHZ	−0.5 (0.9)	−0.8 (1.1)	−0.5 (1.0)	−0.4 (1.0)	0.31 ***	0.15	0.46	0.17 *	0.05	0.3	0.13	−0.06	0.33	0.18	−0.01	0.36
Stunting	61.9	68	55.5	61	−3.98	−15.44	7.48	−2.65	−9.15	3.85	−5.07	−11.78	1.63	−4.24	−10.4	1.93
Underweight	37.1	53.1	30.8	34.9	−16.25 ***	−24.12	−8.38	−7.83 ***	−14.39	−1.26	−8.89	−18.96	1.17	−9.02 ***	−15.1	−2.94
Wasting	4.5	9.3	7	4.9	−9.29 ***	−15.86	−2.72	−2.62	−6.33	1.09	−3.31	−8.2	1.58	−2.47	−5.9	0.95
Boys ^a^																
Height	80.2 (11.2)	80.6 (11.2)	82.4 (11.2)	81.6 (11.8)	−0.05	−1.17	1.06	0.21	−1.31	1.74	0.13	−1.13	1.39	0.22	−0.9	1.35
Weight	10.2 (2.6)	10.2 (2.7)	10.9 (2.8)	10.7 (3.0)	0.17	−0.17	0.52	0.23	−0.11	0.57	0.21	−0.23	0.66	0.25	−0.09	0.6
HAZ	−2.4 (1.3)	−2.6 (1.5)	−2.0 (1.3)	−2.2 (1.4)	0.14	−0.14	0.43	0.16 *	0	0.31	0.08	−0.17	0.33	0.22 *	0.08	0.35
WAZ	−1.7 (1.0)	−2.1 (1.1)	−1.4 (1.1)	−1.6 (1.1)	0.26	0.01	0.51	0.19 **	0.1	0.29	0.17 *	0.01	0.32	0.25 *	0.08	0.42
WHZ	−0.6 (0.9)	−0.9 (1.2)	−0.3 (1.1)	−0.4 (1.0)	0.27 ***	0.08	0.47	0.21 *	0.06	0.36	0.20 *	0.02	0.38	0.21 *	0.07	0.36
Stunting	63.7	65.7	50.8	58.8	0.69	−14.00	15.37	−4.14	−10.48	2.19	−1.27	−10.49	7.95	−6.15 *	−11.76	−0.53
Underweight	37.4	48.8	27.5	34.8	−9.74	−23.38	3.90	−5.03	−11.19	1.13	−3.39	−13.45	6.67	−6.49	−13.15	0.16
Wasting	6.6	15.3 ***	5.9	6.4	−9.55 ***	−14.46	−4.64	−3.11	−6.4	0.19	−3.54	−8.31	1.23	−3.33 *	−6.16	−0.49
<2 years ^b^																
Height	70.0 (6.5)	69.6 (6.7)	70.8 (7.4)	69.2 (7.4)	−0.28	−1.16	0.60	−0.85 *	−1.67	−0.02	−0.91	−2.45	0.63	−0.81 *	−1.6	−0.02
Weight	7.8 (1.5)	7.5 (1.6)	8.1 (1.8)	7.6 (1.8)	0.03	−0.30	0.37	−0.15	−0.38	0.08	−0.17	−0.45	0.11	−0.14	−0.36	0.08
HAZ	−2.0 (1.4)	−2.2 (1.5)	−1.6 (1.4)	−1.9 (1.5)	0.03	−0.21	0.28	0.12	−0.09	0.33	−0.1	−0.37	0.18	0.13	−0.08	0.33
WAZ	−1.5 (1.1)	−2.0 (1.2)	−1.2 (1.2)	−1.6 (1.2)	0.18	−0.04	0.41	0.08	−0.06	0.22	−0.01	−0.24	0.23	0.09	−0.08	0.27
WHZ	−0.6 (0.9)	−1.1 (1.3)	−0.5 (1.1)	−0.7 (1.1)	0.18	−0.04	0.41	0.05	−0.09	0.2	0.1	−0.15	0.34	0.07	−0.08	0.21
Stunting	52	58.2	39.8	50.8	2.76	−5.16	10.68	−2.48	−8.1	3.14	1.61	−6.44	9.66	−3.57	−10.37	3.23
Underweight	32.6	47.1	23.8	37.1	−5.39	−18.43	7.66	−0.46	−7.8	6.89	1.86	−8.42	12.15	−1.24	−8.08	5.6
Wasting	6.7	18.8	6.8	10.3	−9.19 ***	−15.81	−2.57	−1.2	−5.16	2.76	−1.91	−6.88	3.05	−1.03	−4.2	2.13
≥2 years ^b^																
Height	87.1 (7.1)	86.4 (7.9)	88.3 (7.1)	87.9 (7.6)	0.53	−0.12	1.18	0.41	−0.18	1.01	0.59	−0.45	1.63	0.74	−0.16	1.64
Weight	11.7 (1.9)	11.4 (2.0)	12.1 (2.0)	12.1 (2.1)	0.39 ***	0.12	0.66	0.36 ***	0.12	0.6	0.44 **	0.18	0.69	0.44 ***	0.25	0.63
HAZ	−2.6 (1.1)	−2.8 (1.2)	−2.4 (1.1)	−2.4 (1.3)	0.15	−0.02	0.31	0.17 *	0.06	0.28	0.12	−0.03	0.28	0.21 *	0.06	0.35
WAZ	−1.9 (1.0)	−2.1 (1.1)	−1.6 (1.0)	−1.6 (1.0)	0.28 ***	0.12	0.44	0.28 ***	0.18	0.37	0.27 **	0.13	0.41	0.30 ***	0.19	0.41
WHZ	−0.5 (0.9)	−0.6 (1.0)	−0.3 (1.0)	−0.2 (0.9)	0.29 ***	0.11	0.47	0.26 ***	0.17	0.35	0.29 **	0.12	0.46	0.27 ***	0.14	0.4
Stunting	73	73.1	62.8	65.8	0.05	−6.01	6.11	−4.82	−10.23	0.6	−4.05	−12.54	4.44	−6.66 **	−12.13	−1.18
Underweight	41.5	53.3	32.8	33.3	−14.87 ***	−23.27	−6.46	−10.45 ***	−16.02	−4.88	−9.2	−18.52	0.11	−11.40 ***	−16.66	−6.13
Wasting	4.9	8.2	6.1	2.7	−8.51 ***	−13.91	−3.11	−3.86 **	−5.98	−1.74	−6.22 **	−9.22	−3.22	−4.10 **	−6.43	−1.78
All ^c^																
Height	78.9 (10.9)	79.3 (11.1)	80.8 (11.3)	80.3 (11.9)	0.11	−0.51	0.72	0.42	−0.68	1.52	−0.11	−1.08	0.86	0.48	−0.33	1.28
Weight	9.8 (2.6)	9.8 (2.7)	10.4 (2.8)	10.3 (3.0)	0.26 **	0.05	0.47	0.27 *	0	0.55	0.17	−0.12	0.47	0.29	−0.01	0.6
HAZ	−2.3 (1.3)	−2.6 (1.4)	−2.1 (1.3)	−2.2 (1.4)	0.17 *	0.03	0.31	0.14 *	0.03	0.25	0.05	−0.12	0.23	0.18 *	0.09	0.27
WAZ	−1.7 (1.0)	−2.1 (1.1)	−1.4 (1.1)	−1.6 (1.1)	0.29 ***	0.15	0.44	0.19 **	0.11	0.28	0.18 *	0.07	0.29	0.22 **	0.15	0.29
WHZ	−0.5 (0.9)	−0.8 (1.1)	−0.4 (1.1)	−0.4 (1.0)	0.29 ***	0.15	0.42	0.18 *	0.09	0.28	0.24 *	0.08	0.4	0.19 *	0.09	0.3
Stunting	63	66.7	52.9	59.8	−1.34	−7.12	4.44	−3.51	−7.83	0.82	−2.18	−10.22	5.87	−5.16 *	−9.55	−0.77
Underweight	37.3	50.7	28.9	34.8	−12.54 ***	−19.82	−5.25	−6.29 ***	−10.96	−1.62	−5.19	−10.75	0.37	−7.35 ***	−11.62	−3.08
Wasting	5.8	12.7	6.4	5.7	−9.32 ***	−14.86	−3.79	−2.86 *	−4.91	−0.8	−4.84 ***	−8.62	−1.06	−2.84 **	−5.58	−0.1

* *p* < 0.05; ** *p* < 0.01; *** *p* < 0.001. ADID = Adjusted difference-in-differences. ^a^ Adjusted for father’s educational attainment, household wealth index, child age, caste/ethnicity, and family size; weighted with bootstrapping; ^b^ Adjusted for father’s educational attainment, household wealth index, caste/ethnicity, gender, and family size, weighted with bootstrapping; ^c^ Adjusted for father’s educational attainment, household wealth index, caste/ethnicity, gender, child age in month, and family size, weighted with bootstrapping. # Radius = 0.02; ! Weighted with bootstrapping. Z scores for height-for-age (HAZ), weight-for-age (WAZ) and weight-height (WHZ).

We apologize for any inconvenience caused to the readers by this error. 

## References

[1] Renzaho A.M.N., Chitekwe S., Chen W., Rijal S., Dhakal T., Dahal P. (2017). The Synergetic Effect of Cash Transfers for Families, Child Sensitive Social Protection Programs, and Capacity Building for Effective Social Protection on Children’s Nutritional Status in Nepal. Int. J. Environ. Res. Public Health.

